# Spirituality, depression and quality of life in medical students in KwaZulu-Natal

**DOI:** 10.4102/sajpsychiatry.v22i1.731

**Published:** 2016-03-22

**Authors:** Narushni Pillay, Suvira Ramlall, Jonathan K. Burns

**Affiliations:** 1 Department of Psychiatry, Nelson R. Mandela School of Medicine, University of KwaZulu-Natal, South Africa

## Abstract

**Background:**

The majority of studies on spirituality demonstrate its positive association with mental health. Despite the increasing number of studies, there remains a dearth of studies emanating from African countries looking at the relationship between mental illness, quality of life and measures of spirituality. The present study evaluates the role of spirituality in relation to current depression and quality of life in medical students, who are known to be at high risk for depression.

**Objectives:**

The aim of this study was to determine the prevalence of moderate and severe depressive symptoms in this population and explore potential correlations between spirituality, depression and quality of life.

**Methods:**

230 medical students were surveyed at the University of KwaZulu-Natal Medical School, using the Zung Self-Rating Depression Scale (Zung SDS), Spiritual Involvement and Beliefs Scale (SIBS), WHO Quality of Life Scale (WHOQOL) and a demographic data sheet.

**Results:**

There was a high prevalence of depressive symptoms in the medical students, with a significant proportion (15.6%) showing evidence of severe depressive symptoms (indicating likely depressive illness). Those with a history of mental illness or of having attended traditional, complementary or alternate medical practitioners showed higher levels of depression. Lower spirituality was associated with non-adherence to a major religion and a history of mental illness. Quality of life was better in second and fifth year students and poorer in those with a history of mental illness.

**Conclusion:**

Medical students’ experiences of depression (most probably due to stress) and its relationship with spirituality and quality of life merit further investigation with a view to establishing policy guidelines for dealing with this issue.

## Introduction and background

Over the last 25 years, spirituality has become a topic of increasing interest to medical professionals in the field of mental health.^1,2^ While much research initially focused on general medical disorders, the role of spirituality in mental and substance-related disorders has been the focus of a growing literature base.^1,2^ Key to any research in the field of spirituality is the definition used. From the Vedic definition of spirituality as a personal connection with God or a supreme being to the more clinical definition of spirituality as a journey towards transcendental consciousness and connectedness with one’s own inner life force, spirituality is not uncommonly used interchangeably with religion.^3^ Religion, however, is considered to focus more on structured beliefs, theology, religious traditions and codes of conduct.^2,3^ The difficulty in isolating spirituality as a valid construct has given rise to concerns about how it is measured.^4^ Admittedly, there is an overlap between the two concepts and in this study the two terms were used interchangeably, acknowledging that one can be spiritual without being affiliated with any religious organisation and vice versa.

Spirituality has been shown to influence quality of life.^5^ Quality of life is a broad concept and a measure of overall well-being and the individual’s perception of their position in life, including the aspects of physical and mental health, relationships and environment.^5^ The connection between spirituality and quality of life is thought to exist through the areas of mental health, social support (including support from the religious community) and improved health (as prescribed by certain religions).^5^ The World Health Organization (WHO) also includes spirituality in its quality of life assessments.^6^

A strong scientific connection has been made between spirituality and general medicine in the literature, and this has influenced practice, for example in integrating pastoral care in hospitals as part of patient management.^7^ In psychiatry, there is evidence of a positive association between spirituality and treatment outcomes for depression, anxiety, substance abuse and suicide.^8^ Multiple studies, both longitudinal and cross-sectional, have shown that spirituality has an unequivocal positive effect against depression, influencing response to treatment and significantly reducing prevalence and incidence rates.^7,8^ However, in contrast, it has also been argued that an awareness of religious and spiritual activities of patients can show both their value as a resource for healthy mental and social functioning, as well as an indication of when such beliefs are distorted and limiting, and contribute to pathology rather than alleviate it.^9^

Depression, a widely prevalent mental health problem, is the fourth principal cause of disease affliction and the leading cause of non-fatal burden, affecting 12% of the population.^10^ In the South African Stress and Health (SASH) study, it was found that the lifetime prevalence for major depression was 9.7%.^11^ The WHO projects depression to be the second leading cause of burden of illness by the year 2020.^10^ Given the current and anticipated increased burden of depression and its consequent cost implications, identifying preventive strategies will positively impact outcomes.

There are many theories regarding the causes of depression, which have been well-documented.^12^ These include the bio-psycho-social-spiritual model and the diathesis-stress model.^13^ Despite criticisms of the former for being theoretically weak, simplistic and polarising,^14,15^ and the latter for being problematic in isolating predisposition within the context of environmental susceptibility,^16,17^ both of these models identify stress as being a contributing factor to depression. Further, depression is negatively associated with quality of life, while spirituality is positively associated with quality of life.^5^ Demonstration of a possible protective effect of spirituality on depression will therefore support its endorsement as a strategy to increase resilience.

Medical school is well established as being a high-stress environment, and depression is prevalent in medical students.^18^ Between 1980 and 2005, 40 studies investigated depression in medical students, reporting prevalence rates of 13–26%.^18^ Further, medical students are noted to have higher levels of depression than their non-medical peers, thus identifying them as a vulnerable student population.^18^ However, several studies have demonstrated a significant decrease in depressive symptoms and an increase in quality of life in medical students in association with spirituality in medical students.^18^

The majority of available literature on spirituality focuses on studies carried out in the US and UK, with a significant lack of research focusing on the assessment of spirituality and quality of life in African or Eastern countries.^1,2^ Furthermore, research populations have been predominantly of Judeo-Christian religions.^1,2^ In South Africa, there is a small but growing literature on spirituality, all concurring that spirituality must be integrated into the local practice of psychiatry; however, there have not been studies measuring these constructs within the South African context.^7,8^ Given the burden of depression and stress associated with medical students, this study was therefore embarked upon to explore potential associations between spirituality, depression and quality of life in medical students attending the University of KwaZulu-Natal.

## Method

### Participants

A cross-sectional convenience sample of medical students attending the Nelson R Mandela School of Medicine at the University of KwaZulu-Natal were surveyed using four self-administered questionnaires. All 950 students from first to final year were eligible to participate in the study. There were no exclusion criteria. Participation was voluntary and written informed consent was required. Confidentiality was maintained and questionnaires were submitted anonymously.

### Instruments

The following tools were employed: a demographic data sheet, the Zung self-rating depression scale (Zung SDS), the Spiritual Involvement and Beliefs Scale (SIBS) and the WHO Quality of Life Scale (WHOQOL).

#### The demographic data sheet

This included year of study, age, race, gender, current medical and psychiatric history, socio-economic status (income), religious affiliation, traditional beliefs and consultation with traditional, complementary and alternate health practitioners.

#### The Zung self-rating depression scale (Zung SDS)

This is a reliable and valid tool to screen for and rate depression.^19,20^ Strong significant correlations have been found between the Zung SDS and the Hamilton Rating Scale for Depression, as well as with the Minnesota Multiphasic Personality Inventory Depression Scale.^20^ The Zung SDS contains 20 statements and is scored on a Likert scale.^19^ Studies differ on the cut-off values for screening for depression, ranging from scores of 40 to 55.^19^ According to Zung, the majority of depressed individuals score between 50 and 69, with 80 being the highest possible score.^19^ For this study, scores above 50 were taken as indicative of a diagnosis of current depression while scores between 30 and 50 were regarded as indicating the presence of depressive symptoms.

#### The Spiritual Involvement and Beliefs Scale (SIBS)

This is a multi-dimensional scale that was devised to be broadly appropriate and valid across religious conventions in assessing values, attitudes and behaviours.^21^ It has been found to be reliable, with high validity,^21^ although it has been suggested elsewhere that its validation may still be preliminary.^22^ It has a strong correlation with the Spiritual Well-Being Scale, another recognised measure of spirituality.^21^ The SIBS comprises 26 questions in a modified Likert-type format using an ordinal scale of ‘strongly agree’, ‘agree’, ‘neutral’, ‘disagree’ or ‘strongly disagree’.^21^ The items encompass four domains, namely internal viewpoints, external activities, personal application and existential and meditative ideas.^21^ The total score is 130.^21^ The scoring has a linear relationship with spiritual beliefs, with a higher score having a proportionally higher involvement in spirituality.^21^

#### The World Health Organization Quality of Life (WHOQOL) questionnaire

This tool is a measure of quality of life and has proved valuable in quality of life studies.^23^ It has also been validated for study in medical students.^23^ It contains 26 questions in a five-point Likert format, containing four domains, namely physical, psychological, social and environmental, with each score denoting the individual’s perception of quality of life in each domain.^23^ Domain scores are scaled positively: a higher score denotes a higher quality of life.^23^ Since the SIBS was used to measure spirituality, this research did not use the ‘Spirituality, Religion and Personal Beliefs’ addition to the WHOQOL. This was also because the inclusion of ‘positive psychology’ items such as positive or negative feelings, or components of well-being and distress, as part of the definition of spirituality, would be tautologous, and, hence limiting.^4^

#### Ethical considerations

Ethical approval for this study to proceed was obtained from the University of KwaZulu-Natal’s Biomedical Research Ethics Committee (Ref BE178/13).

### Data analysis

Of the 950 medical students, from first to fifth year, 646 (68.0%) declined. 304 (32.0%) agreed to participate and 74 (24.3%) sets of questionnaires were incomplete and excluded from the final analysis. The final sample comprised 230 (24.2%) medical students. All information was captured in Microsoft Excel and subsequently analysed descriptively on the spreadsheet while further statistical computations used the Statistical Package for Social Sciences (SPSS version 19).

All demographic data were analysed descriptively and presented as frequency counts. As all the instruments yielded ordinal data, the median was used to summarise these measures. Further statistical computations used non-parametric techniques. The Mann-Whitney U-test was used for all two-group comparisons, and the Kruskal-Wallis test was used for comparisons involving three or more groups. The Spearman Rank Order Correlation was used for analysing the relationships between the Zung SDS, SIBS and WHOQOL, and the Zung SDS and WHOQOL domains. The alpha level was set at 0.05, so any *p*-value less than 0.05 would be considered to be statistically significant.

## Results

A total of 230 medical students participated in this research. The average age was 21 years and there were more women (71.3%) than men (28.7%). The proportion of participants from each year of study was highest at first year level (28.2%) and decreased in a linear fashion through second year (22.2%), third year (20.4%), fourth year (16.5%) and fifth year (12.0%). Ethnic (racial) representation was predominantly black (64.3%), followed by Asian (25.2%), with relatively few mixed-race (6.1%) and white (4.3%) participants. The main religion was Christian (77.8%), followed by Hindu (16.1%), with very few Muslim (2.6%) and Other (3.5%). Most participants were in the middle R15 000–R25 000 per month income bracket (60.0%), followed by the less than R15 000 per month bracket (30.9%) and the higher over R25 000 per month bracket (9.1%). Just over half (53.9%) lived in urban areas, just under a third in rural areas (30.0%) and the remainder in peri-urban areas (16.1%). Other background information indicated current mental illness (3.0%), other current medical conditions (8.3%), substance abuse (33.9%), presence of traditional beliefs (50.4%) and consultation with any traditional, complementary or alternate medical practitioner (TCAMP) (20.0%). Demographic traits are compiled in Table 1 together with the median scores on each instrument.

**TABLE 1:** Demographics and median scores on each instrument

Demographic characteristic	*n* (%)	Instrument					

		Zung SDS		SIBS		WHOQOL	
		
		Median	*p*-value	Median	*p*-value	Median	*p*-value
**Year of study**							
1	65 (28.2)	34	0.296	99	0.905	6	0.000*
2	51 (22.2)	34	-	100	-	7	-
3	47 (20.4)	35	-	98	-	6	-
4	38 (16.5)	40	-	100.5	-	6	-
5	29 (12.6)	35	-	99	-	7	-
**Age**							
Range = 18–32 years							
Mean = 21							
**Race**							
Asian	58 (25.2)	34.0	0.220	100.5	0.398	6	0.686
Black	148 (64.3)	37.0	-	99.5	-	6	-
Mixed-race	14 (6.1)	34.5	-	97.0	-	6	-
White	10 (4.3)	33.0	-	92.0	-	6	-
**Gender**							
Female	164 (71.3)	37	0.066	99	0.532	6	0.723
Male	66 (28.7)	34	-	100	-	6	-
**Religion**							
Christian	179 (77.8)	36	0.303	100.0	0.007*	6.0	0.213
Hindu	37 (16.1)	34	-	100.5	-	6.0	-
Muslim	6 (2.6)	43	-	100.0	-	5.5	-
Other	8 (3.5)	36	-	69.0	-	6.0	-
**Household income per month**							
< R15 000	71 (30.9)	36	0.440	99	0.790	6	0.515
R15 000–R25 000	138 (60.0)	35	-	99	-	6	-
> R25 000	21 (9.1)	36	-	99	-	6	-
**Region**							
Peri-urban	37 (16.1)	38.0	0.481	102	0.466	6	0.213
Rural	69 (30.0)	36.0	-	99	-	6	-
Urban	124 (53.9)	34.5	-	99	-	6	-
**Mental illness**							
No	223 (97.0)	36	0.024*	100	0.018*	6	0.002*
Yes	7 (3.0)	54	-	92	-	5	-
**Medical history**							
No	211 (91.7)	35	0.077	99	0.302	6	0.171
Yes	19 (8.3)	42	-	99	-	6	-
**Substance abuse**							
No	152 (66.1)	35	0.802	99	0.078	6	0.654
Yes	78 (33.9)	36	-	100	-	6	-
**Presence of traditional belief**							
No	114 (49.6)	35.0	0.328	100	0.908	6	0.599
Yes	116 (50.4)	36.5	-	99	-	6	-
**Consultation with any traditional, complementary or alternate medical practitioner (TCAMP)**							
No	184 (80.0)	35.0	0.040*	99	0.285	6	0.107
Yes	46 (20.0)	38.5	-	99	-	6	-

*Source:* Author’s own work.

Comparisons within the various demographic characteristics (also summarised in Table 1) indicated a few significant differences between groups on some of the instrument scores. Spirituality scores (SIBS) were significantly higher in those indicating consultation with any TCAMP and significantly lower in those whose religion was specified as ‘Other’, although this latter result needs to be regarded with caution as this only involved eight participants. There was no difference in either depression scores or spirituality scores between different years of study; however, quality of life was perceived to be better in the second and fifth year students compared to the other years (*p* < 0.000). Finally, the seven students (3.0%) who indicated a current mental illness all had significantly higher depression levels, lower levels of spirituality and perceived a poorer quality of life. Again, this result needs to be regarded with caution because of the small sample size.

The prevalence of moderate and severe depressive symptoms among the medical students may be seen in Table 2. These results show that almost three-quarters of the medical students (76.5%) who participated in this research were experiencing problems with depressive symptoms, with one-fifth of these (15.6% of total sample) having severe depressive symptoms. Notably this means only a quarter of students were experiencing minimal or no symptoms of depression.

**TABLE 2:** Prevalence of depressive symptoms.

Score and Mean	Score and Mean	Score and Mean
Zung SDS score less than 30	Zung SDS score between 30 to 50	Zung SDS score over 50
(Minimal depressive symptoms)	(Moderate depressive symptoms)	(Severe depressive symptoms)
*n* = 54 (23.5%)	*n* = 130 (60.9%)	*n* = 36 (15.6%)

*Source*: Author’s own work.

Table 3 reports the results of the correlational analysis performed between the ZUNG SDS, SIBS and WHOQOL scores.

**TABLE 3:** Correlation coefficients for Zung SDS, SIBS and WHOQOL.

Variable	Correlation coefficient (*ρ*)	*p*-value
**Overall correlations**	
Zung SDS with SIBS	–0.143	0.030*
Zung SDS with WHOQOL	–0.483	0.000*
WHOQOL with SIBS	0.294	0.000*
**Correlations of Zung SDS with WHOQOL domains**	
Zung SDS with WHOQOL physical	–0.373	0.000*
Zung SDS with WHOQOL psychological	–0.468	0.000*
Zung SDS with WHOQOL social	–0.221	0.001*
Zung SDS with WHOQOL environmental	–0.214	0.001*

*Source*: Author’s own work.

The analysis yielded a significant negative correlation (*ρ* = –0.143) between depressive and spirituality scores (*t*(228) = 2.188; *p* = 0.030), a significant negative correlation (*ρ* = –0.483) for depressive symptoms and quality of life (*t*(228) = 8.331; *p* = 0.000), and a significant positive correlation (*ρ* = 0.294) between spirituality and quality of life (*t*(228) = 4.642; *p* = 0.000). Thus, higher levels of depression were associated with lower levels of spirituality and poorer quality of life. Higher levels of spirituality were associated with perceived better quality of life. When looking at the association between depression and individual domains of quality of life, the Zung SDS had a significant inverse association with all of the WHOQOL domains.

Taken together, these results have shown some associations between certain demographic variables and the scores on one or more of the instruments used. In particular, those with a history of mental illness or of having consulted TCAMPs showed higher levels of depression. Lower spirituality was associated with non-adherence to a major religion and a history of mental illness. Quality of life was better in second and fifth year students and poorer in those with a history of mental illness. Depressive symptoms were present in almost three-quarters of the medical students, with a significant proportion showing evidence of a severe depressive symptoms (indicating likely depressive illness).

## Discussion

To the best of our knowledge, this is the first study examining the rates of depressive symptoms in medical students in KwaZulu-Natal, investigating relationships between depression, spirituality and quality of life. While this was a small-scale study, it has shed light on these aspects as they affect medical students.

The finding of elevated rates of depressive symptoms among the current sample of medical students confirms trends evidenced in previous studies.^18^ While one could argue that the post-apartheid context in South Africa should be associated with decreased depressive symptoms due to decrease in stressors, such as freedom to study for all race groups at any level in South Africa with fewer social, political and economic restrictions, there are still many stressors facing medical students, such as academic pressure, personal issues, living alone for the first time, their developmental stage, taking on adult responsibilities, studying for exams and fear of job security. In particular, this study may have something to say about the process of transformation at university level. This is particularly relevant to first year students with respect to ‘underprepared’ students with poor schooling backgrounds who face many challenges in adapting to university study.^24^ In addition, with the increase in study years, students need to travel to different hospitals, consulting with many more patients. Many students also face financial challenges, such as the expenses of books, transport and food; close to one-third of the current sample fell into the family income of less than R15 000 per month bracket. Finally, there is ongoing stress from societal expectations of medical students.

Of particular interest with respect to depression was consultation with any TCAMP: students showed higher depressive symptoms. It is hypothesised that this could possibly be due to help-seeking behaviour to deal with depressive symptoms. It is interesting to note the inclusive role of holistic healing where it can be seen that help-seeking has not been limited only to general practitioners or specialist psychiatrists.

The negative relationship between spirituality and depressive symptoms supports the findings of previous studies.^25^ With respect to spirituality, the South African population is generally pious, which was evident in this study. To deal with stress, some students turn to spirituality or a higher force to cope with feelings of hopelessness. Belief in a higher power can be seen to be helping define one’s purpose in life. This would be linked to perceived quality of life; this research showed lower depressive symptoms and higher levels of spirituality to be associated with a better quality of life, especially in the psychological domain. Generally, with a higher degree of spirituality comes a higher sense of well-being^5^ and therefore a better quality of life.

The role of spirituality is also particularly important in the training of psychiatrists. While this domain was ignored for many years, more recent research has suggested that spirituality should be integrated into the practical training of psychiatrists, especially in a diverse setting like South Africa which is multi-cultural, with many religions being practised.^26^ An awareness of their own spirituality would also help clinicians in providing more holistic care for their patients.^27^

Finally, conceptually, this study chose to use religion and spirituality interchangeably. Future research should consider separating these concepts and tightening their definitions as it has been argued recently that spirituality is a much broader concept which should not be quantified only through the lens of religion.^26^

Limitations foreseen were incomplete filling in of information leading to inadequate data. This was overcome by obtaining a proper sample size. The voluntary participation may have introduced bias as depressed individuals might not want to participate, being influenced by depressive symptoms, for example lack of energy and decreased concentration. There is the possibility that some of the results were falsified, leading to under-reporting, as medical students have some knowledge of depression and want to avoid the stigma of mental illness. This was overcome by allowing anonymity. Another limitation that may be noted is the inherent limitations of any self-reported measures. Finally, the cross-sectional nature of this study does not allow us to determine causal relationships between depression, spirituality and quality of life.

## Conclusion

This study has clearly shown that depression among medical students is a matter for concern which needs to be addressed. In developing a healthcare system in South Africa designed to serve everyone, medical students are an important population. Given the negative association of depression with spirituality, it is recommended that facilities for counselling and the provision of spiritual outlets for support should be included in government policies to work towards decreasing depressive symptoms in medical students. It is necessary to address the unhappiness related to the poorer perceived quality of life so as to facilitate the retention of professional services in South Africa.
